# SV40 Large T Antigen Is Not Responsible for the Loss of STING in 293T Cells but Can Inhibit cGAS-STING Interferon Induction

**DOI:** 10.3390/v12020137

**Published:** 2020-01-24

**Authors:** Joshua B. Reus, Guillermo S. Trivino-Soto, Lily I. Wu, Kristiana Kokott, Efrem S. Lim

**Affiliations:** 1 Center for Fundamental and Applied Microbiomics, Biodesign Institute, Arizona State University, Tempe, AZ 85287, USA; 2 School of Life Sciences, Arizona State University, Tempe, AZ 85287, USA

**Keywords:** SV40 polyomavirus, large T antigen, cGAS, STING, interferon, 293T

## Abstract

Several DNA viruses have evolved antagonists to inhibit the cyclic GMP–AMP synthase (cGAS)-stimulator of interferon genes (STING) DNA-sensing immune pathway. This includes DNA viral oncogenes that antagonize the cGAS-STING pathway by binding STING through the LxCxE motif. The 293T human cells are widely used in biology studies as they are highly transfectable. While parental 293 cells express high levels of STING, 293T cells lack STING and are unable to induce interferon antiviral responses to cytosolic DNA. Additionally, 293T cells express the SV40 polyomavirus large T antigen (LT) which enhances the replication of transfected DNA plasmids carrying the SV40 origin of replication. Since SV40 LT also encodes the LxCxE motif, the lack of STING expression in 293T cells is commonly assumed to be due to SV40 large T antigen. We find that SV40 LT does not alter exogenously expressed and endogenous levels of STING protein. We show that STING transcription is suppressed in 293T cells but is not driven by SV40. This study also revealed that SV40 LT does indeed inhibit cGAS-STING interferon induction, but through a mechanism distinct from other DNA virus oncogenes. Collectively, these results indicate that while SV40 LT can inhibit cGAS-STING interferon induction, it does so in an unanticipated manner.

## 1. Introduction

Immortalized cell lines engineered with viral genes are commonly used in biology studies. The human embryonic kidney 293 cells were transformed with human adenovirus 5 DNA including the E1 gene that prevents apoptosis and dysregulates the cell cycle [1]. The 293T cells, a cell line widely used in virology and cellular biology experiments, were derived from the 293 cell line with the stable expression of SV40 polyomavirus large T antigen (LT) [2]. The multi-functional SV40 LT regulates viral transcription and genomic replication by binding to the SV40 origin of replication (SV40 Ori) sequence [3,4]. Cells expressing SV40 LT are also able to replicate transfected DNA plasmids that contain SV40 Ori to high copy numbers. Additionally, 293T cells are deficient in the cyclic GMP–AMP synthase (cGAS)-stimulator of interferon genes (STING) DNA-sensing immune pathway making them highly transfectable with DNA plasmids [5,6,7].

DNA sensing through the cGAS-STING pathway is an important part of the innate immune defense [8]. Activation of cyclic GMP–AMP synthase (cGAS) and the downstream regulator stimulator of interferon genes (STING) leads to a signaling cascade mediated by TANK-binding kinase 1 (TBK1) and interferon regulatory factor 3 (IRF3), culminating in the induction of type I interferons [9]. Given the potent selective pressures exerted by these host antiviral responses, viruses have evolved strategies to antagonize the cGAS-STING pathway [10,11]. For example, DNA tumor virus oncoproteins adenovirus E1A and human papillomavirus E7 bind STING preventing the cGAS-STING interferon activation [12]. The LxCxE motif involved in binding to retinoblastoma protein (Rb) is also essential for STING binding [12,13]. Dengue virus NS2B3 protease directly cleaves STING preventing phosphorylation of IRF3 [14,15]. Kaposi’s sarcoma-associated herpesvirus (KSHV) vIRF1 protein inhibits interferon-beta production by blocking the TBK1 activation by STING [16]. 

Like adenovirus and human papillomavirus, polyomavirus LT proteins also contain the conserved LxCxE motif which binds and inhibits Rb. Hence, the lack of STING expression in 293T cells is assumed to be due to SV40 LT directly interacting with STING through the LxCxE motif. In this study, we report that SV40 LT inhibits cGAS-STING interferon induction through a mechanism distinct from other viral oncogenes and uncover an unanticipated defect underlying the loss of STING in 293T cells. 

## 2. Materials and Methods

### 2.1. Cell Culture

The 293 (ATCC), 293T (ATCC), 293TT (NCI), and HeLa (ATCC) cells were maintained in DMEM supplemented with 10% fetal bovine serum, and 1% penicillin/streptomycin at 37 °C in 5% CO_2_. Additionally, 293TT cells were maintained with 250 μg/mL of Hygromycin B. A separate source of 293 cells, a generous gift from Dr. Brenda Hogue, was used to independently verify the experiments. Human Renal Proximal Tubule Epithelial Cells (HRPTEC) were maintained in Renal Epithelial Basal Medium (ATCC) supplemented with 0.5% fetal bovine serum, 1% penicillin/streptomycin, and 2.4 mM l-glutamine. 

### 2.2. Plasmids

SV40 plasmid constructs were generated by PCR amplification of the early region (Fw 5’-CGCCGGCGATAAAGTTTTAAACAGAGAGG-3’ and Rev 5’-GCGGCCGCTTATGTTTCAGGTTCAGGGGGA-3’) followed by restriction digest cloning into the NotI and NgoMIV sites of the LPCX vector. A hemagglutinin (HA) epitope tag was fused to the N terminus of SV40 constructs. The SV40 large T antigen was cloned from SV40 LT cDNA derived from 293 cells transfected with the SV40 early region plasmid construct using primers (Fw 5’-CGCCGGCGATAAAGTTTTAAACAGAGAGG-3’ and Rev 5’-GCGGCCGCTTATGTTTCAGGTTCAGGGGGAG-3’). The SV40 LT AxAxA mutation was introduced through PCR by performing a series of three PCR reactions. Two initial PCR reactions were performed with either the forward or reverse primer coding for the AxAxA mutation (Fw 5’-AGGAAAACGCGTTTGCCTCAGCAGAAATGC-3’ and 5’-GCATTTCTGCTGAGGCAAACGCGTTTTCCT-3’) with the complimentary primer for archetype SV40 LT. A third reaction combined the PCR products from the first two reactions and used both primers for SV40 LT AxAxA to amplify the mutant gene for SV40 LT. This PCR product was then cloned into the NotI and NgoMIV sites of the LPCX vector. The SV40 small T antigen was cloned from MSCV-N SV40 ST plasmid obtained from Addgene (Cat# 37858). SV40 17kT was cloned from cDNA derived from 293 cells transfected with the SV40 ER plasmid. To generate untagged SV40 ER expression vector, PCR amplification using primers (Fw 5’-AGATCTCCACCATGGATAAAGTTTTAAACAGAGAG-3’) and (Rev 5’-GCGGCCGCTTATGTTTCAGGTTCAGGGGGA-3’) was performed, followed by restriction digest cloning into the BgIII and NotI sites of the LPCX vector. pISRE-luc and pNFκB-luc plasmids were generous gifts from Dr. Grant McFadden. The pUNO1-hSTING-HA3x and pUNO1-hcGAS-HA3x plasmids were purchased from Invivogen. The QuikChange II XL Site-Directed Mutagenesis Kit (Agilent) was used to generate the pUNO1-hSTING^R284S^ (Fw 5’-TTTAGCCGGGAGGATTCTCTTGAGCAGGCCAAA-3’ and Rev 5’-TTTGGCCTGCTCAAGAGAATCCTCCCGGCTAAA-3’) following the manufacturer’s protocol. The STING promoter −803/+139 (Fw 5’-TCAGGTACCGAATGAAATCAAGGCACAGAGCAAG-3’ and Rev 5’-ACGCTCGAGAGCAGGACTCCACACACTCAGCCAA-3’) was cloned into the KpnI and XhoI sites of the pNL1.1 vector (Promega) using enzymes and T4 ligase from NEB. PCR of the coding region of STING was performed using primers for exons 3–6 (Fw 5’-GATGCCCCACTCCAGCCTGCATCCATC-3’ and Rev 5’- CGAATGTTGGGGTCAGCCATACTCAG-3’) and 6–8 (Fw 5’- CTGAGTATGGCTGACCCCAACATTCG-3’ and Rev 5’-AGAGAAATCCGTGCGGAGAGG-3’). STING exon and promoter PCR products were cloned into TOPO-TA vector (ThermoFisher Scientific). All cloned plasmid constructs were verified by Sanger sequencing and western blot analysis for protein expression.

### 2.3. Transfection

The 293 cells were seeded a day prior to transfection at a density of 1 × 10^5^ cells/mL in 1 mL of complete growth medium in each well of a 12-well tissue culture plate. DNA plasmids were incubated in serum-free DMEM complexed with TransIT-LT1 transfection reagent at a ratio of 1:3 for 15 min, and then added to cells. Equal total amounts of DNA were used in each transfected well by increasing total DNA with the appropriate empty vector plasmids. For luciferase assays, cells were seeded at 2 × 10^4^ cells/mL in a 96-well plate. NanoLuc luciferase reporter plasmids were co-transfected with a firefly control plasmid under the control of a TK promoter (pGL4.54[luc2/TK]).

### 2.4. Western Blotting

At 24 hours post-transfection, growth media was aspirated, cells were carefully washed with PBS, and subsequently lysed with Cell Extraction Buffer (Life Tech) with added Pierce protease and phosphatase inhibitors. The lysates were incubated on ice for 15 min followed by centrifugation for 5 min at ≥8000 rcf and 4 °C. LDS Sample Buffer was added to cell lysates at a final concentration of 1× followed by incubation at 100 °C for 5 min. The lysates were then subjected to SDS-PAGE on NuPAGE™ 4%–12% Bis-Tris precast Protein Gels (Invitrogen). Proteins were then transferred from SDS-PAGE gel to polyvinylidene fluoride membrane and detected using the following primary antibodies: rabbit anti-STING (1:2000; Cell Signaling), rabbit anti-TBK1 (1:1000; Cell Signaling), and rabbit anti-IRF3 (1:1000; Cell Signaling), rabbit anti-cGAS (1:2000; Millipore), mouse anti-HA.11 (1:3000, Biolegend), monoclonal SV40 T-antigen antibody (1:1000 dilution; Pab416, Santa Cruz Biotech). Secondary antibodies used: anti-mouse IgGκ HRP (1:3000, 1:5000 for anti-HA.11; Santa Cruz Biotech) and mouse anti-rabbit HRP (1:3000; Santa Cruz Biotech). SuperSignal™ West Femto Maximum Sensitivity Substrate (Thermo Scientific) was used to visualize proteins following secondary antibody incubation.

### 2.5. Quantitative Reverse-Transcription PCR 

All cell lysis steps for RNA extraction were performed with reagents that had been chilled on ice prior to collection (PBS and lysis buffer). QIAshredder columns were used to homogenize cell lysates before the extraction of total RNA following the manufacturer’s protocol for the RNeasy mini kit (Qiagen). Quantification of mRNAs was carried out by first-strand synthesis using the Superscript III First-Strand System (Invitrogen) following the included protocol for synthesis with oligo(dT)_20_ primers. Following cDNA synthesis, primers were added to Applied Biosystems fast SYBR green master mix at a final concentration of 300 nM for the forward and reverse primer. The primers used are as follows: Interferon-β (FW 5’-AGGACAGGATGAACTTTGAC-3’ and Rev 5’-TGATAGACATTAGCCAGGAG-3’), RSAD2 (FW 5’-GTGAGCAATGGAAGCCTGATC-3’ and Rev 5’-GCTGTCACAGGAGATAGCGAGAA-3’), and GAPDH (FW 5’-GCACCGTCAAGGCTGAGAAC-3’ and Rev 5’-ATGGTGGTGAAGACGCCAGT-3’). STING mRNA (Fw 5’-GCTCCAGGCCCGGATTCGAAC-3’ and Rev 5’-CCTATCCTCCCGGCTAAAGCC-3’) quantification was performed by qRT-PCR using the indicated primers. The qRT-PCR Ct values were converted to absolute copy number using standard curves. Dilutions of the respective control plasmid (5 to 5 × 10^7^ copies) was used to generate a standard curve (*R*^2^ > 0.99). Absolute copies (STING, IFNβ, RSAD2) were normalized to the absolute GAPDH copies for each sample.

### 2.6. Luciferase Assays

For the pISRE-luc and NFκB-luc assays, Bright-Glo (Promega) reagent was used. For the pNL1.1 assay, Dual-Glo (Promega) substrate was used. Cells were transfected for 24 h as described above, lysed, and the appropriate luciferase substrate was added. Promega GloMax Navigator was used to measure luciferase luminescence activity. Student’s *t*-test was used to determine significant differences.

## 3. Results

### 3.1. Differential STING Protein Expression in 293T and 293TT Cells

**Figure 1:**
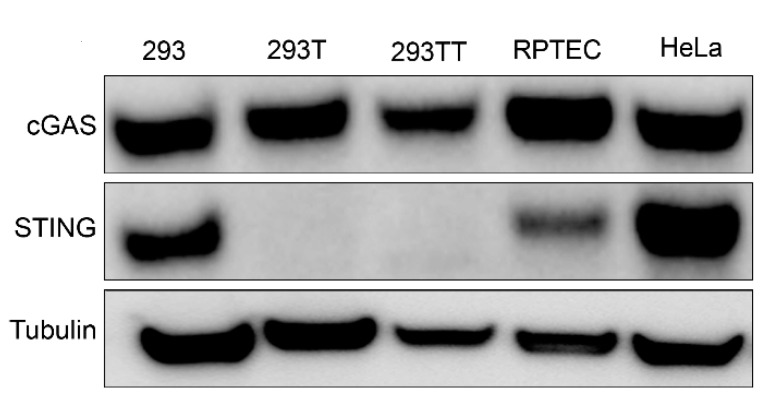
Western blot analysis of endogenous cyclic GMP–AMP synthase (cGAS) and stimulator of interferon genes (STING) expression in a panel of human cell lines. Tubulin was probed as a loading control. Western blot is representative of at least three independent experiments.

While searching for a human cell line that expresses endogenous amounts of cGAS and STING, we found that HEK293-derived cells differentially expressed STING. Western blot analysis showed that 293, HeLa and human primary renal proximal tubule epithelial cells (RPTEC) cells expressed endogenous amounts of cGAS and STING (Figure 1). However, consistent with previous reports [5,7], 293T cells that were derived from 293 cells with the stable expression of SV40 polyomavirus large T antigen did not express STING. 293TT cells, which were generated from 293T cells to further increase expression of SV40 large T antigen [17], also lack STING expression (Figure 1). 

### 3.2. cGAS, STING, and Associated Protein Expression Are not Altered by SV40 LT

**Figure 2:**
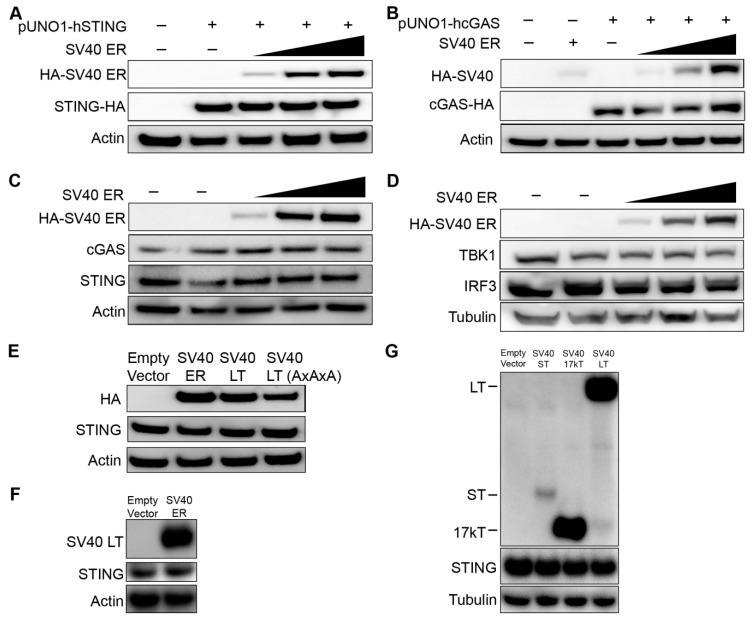
The effect of SV40 early region on exogenous and endogenous cGAS and STING expression. (**A**) The 293T cells were co-transfected with 100 ng of hemagglutinin (HA) epitope-tagged human STING (pUNO1-hSTING-HA3x) with increasing amounts of SV40 early region (ER) plasmid (100 ng, 500 ng, and 1 µg). The anti-HA antibody was used for the detection of SV40 ER and STING. Actin or Tubulin was probed as a loading control as indicated. (**B**) The 293T cells were co-transfected with 100 ng of HA epitope-tagged human cGAS (pUNO1-hcGAS-HA3x) with increasing amounts of SV40 ER plasmid (100 ng, 500 ng, and 1 µg). (**C**,**D**) 293 cells were transfected with the same increasing amounts of SV40 ER plasmid (100 ng, 500 ng, and 1 µg). Western blot analysis for endogenous levels of (C) cGAS and STING, and (D) TANK-binding kinase 1 (TBK1) and interferon regulatory factor 3 (IRF3) was assessed. (**E**) The 293 cells were transfected with 500 ng of SV40 ER, SV40 large T antigen (LT), or SV40 LT AxAxA (mutations in the LxCxE motif). The endogenous expression of STING was assessed by western blot analysis. (**F**) The 293 cells were co-transfected with 1 μg of empty vector or untagged SV40 ER. The figure shows western blot analyses for SV40 LT with a monoclonal SV40 T-antigen antibody, endogenous STING, and Actin (loading control). (**G**) The 293 cells were transfected with 1 µg of SV40 ST, 17kT, or LT plasmid. Western blot analysis was performed at 24 hours post-transfection for endogenous STING expression, HA epitope-tagged SV40 ST, 17kT or LT, and Tubulin (loading control). All western blots shown in this Figure are representative of at least three independent experiments.

We hypothesized that the lack of STING expression could be due to inhibition by SV40 polyomavirus large T antigen expressed in 293T cells. To test this, we co-transfected 293T cells with a plasmid expressing HA-epitope tagged STING with increasing amounts of plasmid encoding the SV40 early region (ER) that expresses the large T antigen through alternative splicing of SV40 ER precursor transcript. We considered that mammalian expression plasmids commonly include the SV40 origin of replication (SV40 Ori) which increases the plasmid replication in cells like 293T that express SV40 large T antigen [18]. To avoid these confounding effects, we used a pUNO1 plasmid lacking the SV40 Ori sequence to express STING (pUNO1-hSTING-HA3x). Western blot analyses of epitope-tagged exogenous STING expression were not altered with increasing amounts of SV40 ER expression (Figure 2A). Exogenously expressed SV40 LT expression levels tested were comparable to the native expression of SV40 LT in 293T cells (data not shown). Although the 293T cells used in our laboratory express cGAS, others report that some 293T cells may not [19]. We performed western blot analyses of epitope-tagged cGAS (pUNO1-hcGAS-HA3x) in co-transfection experiments with increasing amounts of plasmid encoding SV40 ER proteins. SV40 ER proteins did not alter exogenous cGAS expression either (Figure 2B). We next examined whether SV40 ER affected the endogenous expression of cGAS and STING. Endogenous expression of cGAS and STING in 293 cells remain unaltered despite increasing expression of SV40 ER proteins (Figure 2C). TBK1 and IRF-3 proteins involved in the downstream cGAS-STING signaling pathway were also unaffected by SV40 ER (Figure 2D). We verified that endogenous expression of STING was not affected by SV40 LT by repeating the transfection experiments in 293 cells using a plasmid only containing the SV40 LT coding sequence (Figure 2E) and an untagged SV40 ER (Figure 2F). We also examined small T antigen (ST) and 17kT that are produced through alternative splicing of the early region pre-mRNA [20,21], and found that endogenous STING expression was not altered (Figure 2G). These results demonstrate that SV40 large T antigen and SV40 ER proteins do not alter the exogenous or endogenous expression of cGAS and STING. 

### 3.3. Lack of STING Expression in 293T Cells Traces to A Defect in Transcription Regulation, Independent of SV40 LT

**Figure 3:**
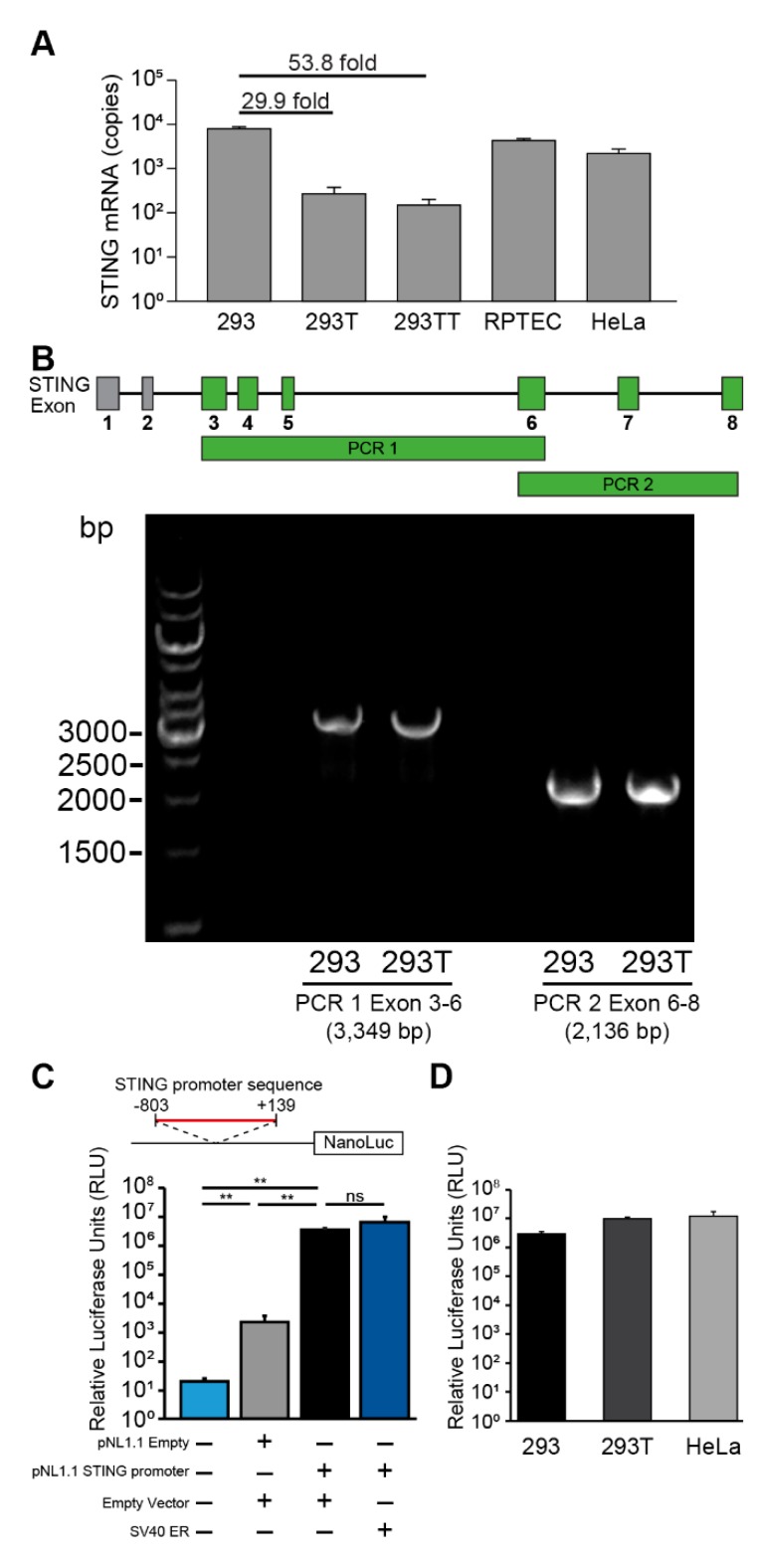
STING transcription is repressed in 293T cells but not in parental 293 cells. (**A**) Quantitative reverse transcription PCR (qRT-PCR) analysis of STING mRNA from the indicated human cell lines is shown. STING mRNA copy numbers were normalized to copies of GAPDH. Absolute copy numbers were determined using validated standard curves. (**B**) The diagram shows the STING coding region and exons 3–8 that encode for the canonical STING protein are highlighted in green. PCR amplification of the STING exons 3–6 (PCR 1, 3349 bp) and exons 6–8 (PCR2, 2136 bp) are indicated. PCR was performed on genomic DNA extracted from 293 and 293T cells. PCR products were separated by electrophoresis on a 0.8% agarose gel. Gel image is representative of three independent experiments. PCR products were cloned into a TOPO vector and Sanger sequenced. (**C**) Luciferase activity of STING promoter-driven luciferase reporter plasmid (90 ng) or the promoterless luciferase control plasmid (100 ng pNL1.1) co-transfected in 293 cells with 100 ng empty LPCX vector or SV40 ER plasmid. The diagram shows the STING promoter sequence cloned upstream of the NanoLuc reporter (Student’s *t*-test; **, *P* < 0.01; ns, not significant). NanoLuc luciferase activity was normalized to control TK firefly luciferase activity (TK promoter pGL4.54[luc2/TK] 10 ng). (**D**) Luciferase activity of STING promoter-driven luciferase reporter plasmid (90 ng) in indicated cell lines. Error bars indicate standard deviations from three replicates. These data are representative of at least three independent experiments.

To understand why 293T cells lack STING protein expression, we quantified STING mRNA transcripts by qRT-PCR. STING mRNA levels were significantly lower in 293T and 293TT cells compared to parental 293 cells (Figure 3A). In contrast, HeLa and RPTEC cells which express STING proteins (Figure 1) had proportionally higher levels of STING mRNA. We next considered whether the DNA coding region for STING might be disrupted in 293T cells. For example, the coding region could be disrupted by the insertion of the SV40 large T antigen gene or other vector sequences. We designed primers spanning the STING coding region (exons 3–8, as the canonical STING protein does not include the sequence-annotated exons 1–2) and amplified it as two overlapping PCR products. Identical PCR products in length (3349 bp and 2136 bp) were obtained after amplifying the genomic DNA from 293 and 293T cells (Figure 3B). To compare the sequences, we cloned the PCR products from both cell lines and performed Sanger sequencing. We verified that the STING coding and intergenic region sequences from 293 and 293T cells were identical and that there were no foreign sequences inserted. This indicates that the lack of STING expression in 293T cells was not due to a disruption of STING coding regions by a sequence insertion event or mutations in splice site sequences.

We next examined the STING promoter sequence (−803 to +139) between 293 and 293T cells and found that their sequences were identical to one another and to published sequences [22,23]. Since there were no mutations between the promoter sequences, we hypothesized that SV40 large T antigen might be inhibiting STING promoter activity. To test whether SV40 large T antigen affects STING promoter activity, we cloned the STING promoter sequence upstream of a NanoLuc luciferase reporter construct (pNL1.1 STING promoter) and co-transfected 293 cells with the STING promoter-luciferase reporter with SV40 ER plasmid. While the STING promoter sequence significantly induced luciferase reporter activity compared to a promoterless luciferase control plasmid (pNL1.1 empty), co-expression of SV40 early region proteins did not alter STING promoter activity (Figure 3C). Finally, we sought to test whether 293T cells expressed other negative regulators of transcription that inhibit STING promoter activity. We reasoned that when controlled for transfection, cell-specific differences in luciferase reporter activity would indicate whether STING promoter activity is differentially regulated between cell lines. However, STING promoter luciferase activity was induced to similar levels across 293, 293T, and HeLa cells (Figure 3D). 

### 3.4. SV40 LT Inhibits cGAS-STING-Mediated Interferon-Beta Induction Independent of the LxCxE Motif

**Figure 4:**
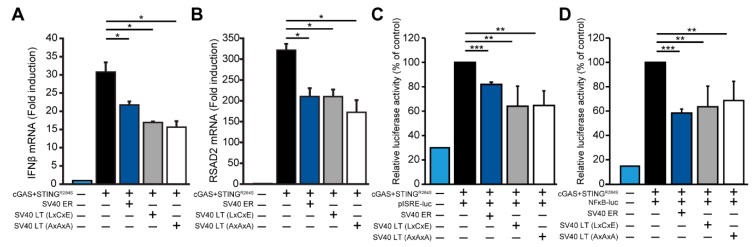
Antagonism of cGAS-STING dependent IFNβ induction by SV40 LT. (**A**) qRT-PCR analysis of 293 cells co-transfected with 100 ng cGAS and 100 ng STING (R284S) with 800 ng of the indicated SV40 plasmid constructs (SV40 ER, LT, or LT AxAxA). Copy number for IFNβ mRNA was normalized to GAPDH and calculated as fold change relative to the control untransfected cells. Error bars indicate standard deviations from three replicates. This data are representative of five independent experiments. (**B**) RSAD2 mRNA copy number from the same experiment in (A) is shown with a similar normalization to GAPDH and expressed as fold change. (**C**) Luciferase activity for pISRE and (**D**) pNFκB reporter constructs of 293 cells co-transfected with cGAS and STING (R284S) with the indicated SV40 plasmid constructs (SV40 ER, LT, or LT AxAxA). Relative luciferase activity was normalized to levels in cGAS-STING induced control cells (first column) (Student’s *t*-test; *, *P* < 0.05; **, *P* < 0.01; ***, *P* < 0.005). Error bars indicate standard deviations from three replicates. This data are representative of at least four independent experiments.

Although SV40 large T antigen does not directly inhibit STING expression, it is possible that SV40 large T antigen may still affect the cGAS-STING interferon induction. We co-transfected 293 cells with cGAS and a constitutively active STING (R284S) [24] and measured interferon-beta induction by qRT-PCR. We found that SV40 ER inhibited cGAS-STING dependent interferon induction (Figure 4A). Moreover, the expression of SV40 large T antigen was sufficient to inhibit the cGAS-STING interferon induction. The ability of adenovirus E1A and papillomavirus E7 to inhibit the cGAS-STING pathway is dependent on the LxCxE motif [12], which is also conserved in SV40 large T antigen. However, mutations in the LxCxE motif did not abrogate the ability of SV40 large T antigen to inhibit cGAS-STING mediated interferon induction (Figure 4A, SV40 large T antigen AxAxA). We validated these findings by measuring RSAD2 (Viperin), an interferon-stimulated gene highly induced by interferon. SV40 ER and large T antigen expression also inhibited cGAS-STING dependent RSAD2 induction (Figure 4B). Additionally, using luciferase reporters for the interferon-stimulated response element (ISRE) and nuclear factor kappa B (NFκB), we found that SV40 large T antigen consistently inhibited cGAS-STING induced ISRE and NFκB activity by approximately two-fold (Figure 4C,D). Thus, SV40 large T antigen inhibits cGAS-STING dependent interferon induction in a modest, but consistent manner independent of the LxCxE motif. 

## 4. Discussion

Our study reveals that SV40 LT is not responsible for the loss of STING expression in 293T cells. First, SV40 LT does not alter cGAS or STING expression in exogenously expressed and endogenous experiments. We tested this using a plasmid encoding the SV40 early region which expresses SV40 LT and other early region proteins through alternative splicing. Second, the loss of STING in 293T cells was concomitant with reduced STING mRNA levels. Parental 293, HeLa and RPTEC cells which expressed higher levels of STING proteins also had relatively higher STING mRNA levels. This indicates that the lack of STING protein expression in 293T was not primarily due to post-translational effects such as protein degradation or cleavage as per other viral antagonists. 

To investigate the reason behind the lack of STING expression in 293T cells, we compared the genomic sequences between 293 and 293T cells. We excluded the possibility of integration by SV40 LT or other vector sequences into the STING coding sequence as they were identical between 293 and 293T cells. The STING promoter sequences from 293 and 293T cells were identical, ruling out mutations in the promoter sequence of 293T cells as an explanation. We also considered whether transcription might be altered by differential expression of transcriptional factors (such as CREB and c-Myc involved in regulating STING transcription) or the presence of a negative regulator (such as SV40 LT) [22]. We considered whether SV40 LT might also have an indirect effect; for example, if SV40 LT altered CREB or c-Myc leading to downstream effects on STING transcription. Transcriptomic studies indicate that up to 136 genes may be differentially regulated between 293 and 293T cells [25]. It is possible that one of these differentially regulated genes may be involved in STING regulation. First, using a plasmid reporter construct, we found that STING promoter activity was not altered by SV40 LT. Second, we took a broader approach—we reasoned that cell-specific differences in transcriptional regulators would be reflected as decreased native STING promoter activity in 293T cells compared to parental 293 cells or other cells that express high levels of STING. However, we found that STING promoter luciferase activity was induced to similarly high levels in 293, 293T, and HeLa cells. This suggests that STING transcriptional regulation in 293T was intrinsically suppressed, but not in parental 293 cells. Epigenetic silencing of STING has been observed in a variety of cancers including colorectal carcinoma and melanoma [26,27]. Moreover, epigenetic reprogramming has been observed in other cell line derivations [28]. Thus, the most parsimonious explanation for impaired STING transcription in 293T cells is an epigenetic process that occurred when 293T cells were derived.

In this study, we uncovered that SV40 large T antigen inhibits cGAS-STING interferon induction. However, SV40 LT inhibition on ISRE and NFκB activity is modest but consistent. This mechanism also differs from adenovirus E1 and human papillomavirus E7 which rely on the LXCXE motif [12]. It is not known whether the ability to inhibit ISRE and NFκB activity is a conserved function of polyomaviruses. BK polyomavirus, a member of the *Polyomaviridae* family closely related to SV40 polyomavirus, evades the innate immune response through an unknown mechanism [29,30]. 

Collectively, these results reveal the underlying cellular consequences of SV40 LT that make 293T cells widely amenable for transfection and other biology studies.
